# Observational study of continuity of HIV care following release from correctional facilities in South Africa

**DOI:** 10.1186/s12889-020-8417-2

**Published:** 2020-03-12

**Authors:** Tonderai Mabuto, Daniel M. Woznica, Gloria Lekubu, Nieser Seatlholo, Nolundi Mshweshwe-Pakela, Salome Charalambous, Christopher J. Hoffmann

**Affiliations:** 1 Aurum Institute, Johannesburg, South Africa; 2 The University of the Witwatersrand School of Public Health, Johannesburg, South Africa; 3 Johns Hopkins University Bloomberg School of Public Health, Baltimore, USA; 4 Department of Correctional Services, Gauteng Region, South Africa; 5 Johns Hopkins University School of Medicine, CRB2 Rm 1M11, 1550 Orleans Rd, Baltimore, MD 21205 USA

**Keywords:** HIV, Linkage to care, Prison, Africa, Antiretroviral therapy

## Abstract

**Background:**

We sought to describe linkage to care, ART continuity, and factors associated with linkage to care among people with HIV following release from incarceration in South Africa.

**Methods:**

We conducted a study of South African correctional service community reentrants who were receiving ART at the time of release. The study was implemented in three of 46 correctional service management areas. Participants were enrolled prior to corrections release and followed up to 90 days post-release to obtain self-reported linkage to care status and number of days of ART provided at corrections release. Clinic electronic and paper charts were sought and abstracted to verify self-reported linkage to care. Log-binomial regression, adjusted for facility, was used to identify associations with post-release linkage to care (self-reported and verified). We sought to specifically assess for associations with HIV diagnosis during index incarceration, ART initiation during index incarceration, and duration of incarceration.

**Results:**

From May 2014 to December 2016, 554 inmates met eligibility and 516 (93%) consented to participate; 391 were released on ART, 40 of whom were excluded from analysis post-release. Of the remaining 351, 301 (86%) were men and the median age was 35 years (interquartile range 30, 40). Linkage to care was self-reported by 227 (64%) and linkage to care could be verified for 121 (34%). At most, 47% of participants had no lapse in ART supply. Initiating ART during the index incarceration showed a trend toward increased self-reported post-release linkage to care. Age > 35 years was associated with increased verified linkage to care while HIV diagnosis outside of a correctional setting and ART initiation during the index incarceration showed trends toward association with increased verified linkage to care.

**Discussion:**

The results of our study are the first description of retention in care following correctional facility release from an African setting and indicate high levels of attrition during the transition from correctional facility to community care. Initiating ART within a correctional facility did not impair post-release linkage to care.

## Introduction

Criminal justice involved populations are recognized to be at risk for HIV infection and, if living with HIV, to have challenges along the HIV care continuum [1]. In many regions of the world, correctional centers concentrate large numbers of persons with HIV or at risk for HIV infection. In southern African correctional facilities, the reported prevalence of HIV infection among inmates ranges from 7.2–35% compared to 4.7–19% for the corresponding country-level general population prevalence [2, 3]. Correctional centers in southern Africa may provide a gateway to HIV treatment and prevention services to a predominantly younger male population, a population that normally exhibits lower engagement in health care [4–7]. Once diagnosed with HIV, inmates in correctional facilities in South Africa are likely to initiate antiretroviral therapy (ART) and achieve favorable treatment outcomes [8, 9].

The proportion of inmates released on ART who link to community clinics post-release is poorly described in South Africa and elsewhere in Africa. There are no descriptions of the potential effect of ART initiation during incarceration on subsequent linkage to care. Most studies on this transition, to date, are from the United States. These studies have reported post-release linkage to care from 28 to 95% with a median reported in a meta-analysis of 36% [10–15]. Differences in care delivery, the criminal justice system, and the social fabric in South Africa compared to the United States may lead to differences in retention in care following corrections release. Thus estimating local linkage to care is important. We sought to estimate linkage to care and ART continuity following release and assess for factors associated with linkage among people on ART leaving correctional facilities in South Africa.

## Methods

### Setting

The study was implemented in the Gauteng Region of South Africa, one of the six administrative regions of the South Africa Department of Correctional Services (DCS). Three management areas (Modderbee, Kgosi Mampuru II, and Baviaanspoort) were selected to provide a range in size of the inmate population, level of security, men and women, those awaiting trial, and sentenced offenders. The daily census across the participating study sites was approximately 14,000 inmates; 29% were awaiting trial. The inmate population in correctional facilities in South Africa is 98% men and 79% black African [16]. Information on the median sentence duration and estimated HIV prevalence in each study facility was not available. All participating facilities had on-site routine ART programs available to all inmates meeting South African national eligibility criteria at the time of the study enrollment (2014–2016). Prior to release, correctional health staff prepared a referral letter for the reentrant to take to a community-based primary health care clinic. In addition, a supply of ART was provided to last between 15 and 90 days [17].

### Study design

Observational study of linkage to care following corrections release.

### Participants

Inmates were eligible to participate if they were receiving HIV-related care at one of the study sites. Additional inclusion criteria were: ≥18 years old, capable of providing informed consent, known or anticipated release date within the study period, anticipated release residence within Gauteng Province, and willingness to provide telephonic or residential contact details to facilitate follow-up activities. In this report we only included participants receiving ART at the time of release. This decision was made because the on-ART population has the most relevance for current South African HIV care models of providing ART to all people with HIV. Study staff based at each of the participating sites worked with the correctional facility health staff and inmate peer educators to recruit and identify inmates who met the eligibility criteria. Those who met eligibility criteria and were interested in participating were provided written and verbal information in their language of choice and enrolled into the study. All participants completed written informed consent prior to enrollment.

This study was conducted according to the principles expressed in the Declaration of Helsinki. Human subject research approval was obtained from the University of the Witwatersrand Human Research Ethics Committee (M120492), the South African Department of Correctional Services Research Review Committee, the Johns Hopkins Medicine Institutional Review Board (NA_83335), and the CDC Institutional Review Board.

### Procedures

Baseline characteristics, including age, sex, duration of incarceration, whether HIV was diagnosed in the correctional facility, and whether ART with initiated in the correctional facility were obtained through structured interviews conducted by research team members while the participant was still incarcerated. The research team members also recorded locator information, including cell phone number, alternative numbers, and location of anticipated residence upon release. Correctional facility health service routine medical records were abstracted onto structured report forms to record the date and result of any CD4 count testing performed closest to the date of release.

Follow-up data collection occurred through structured interviews conducted by research team members. These interviews were conducted either telephonically or in-person. Participants were contacted telephonically at 15 days post-release to verify locator information and starting at 30 days to ascertain post-release care status. These calls were repeated at 60 days and again at 90 days if contact was unsuccessful or linkage not reported during the prior call. The primary purpose of the interviews was to ascertain linkage to care status. Telephonic contact was attempted at least three times, at different times of the day and on different days (including weekends). When telephonic contact was unsuccessful, home visits were attempted in collaboration with DCS community corrections staff. At least three attempts were made to visit the reported place of residence.

Among participants reporting linkage to care, the clinic at which a participant reported receiving care was visited; if no records were located, the participant was contacted again to ask whether they entered care and at which clinic, and to inquire regarding any different names that may have been used to register for care. Records from routine clinical care in public clinics were abstracted to verify self-reported linkage. Local clinics used a mix of electronic care registries, electronic laboratory results, and paper-based charts for patient care and statistical reporting. All three sources were used for verification of self-reported linkage to care. Paper and electronic clinical records were matched to a participant based on name, date of birth, and a range of service dates.

### Measures

#### Outcomes

The primary outcome was the proportion of participants with self-reported linkage to care within 90 days of release. Secondary outcomes were the proportion of participants with verified linkage to care within 90 days of release and the proportion of participants without a lapse in ART possession following release.

- Self-reported linkage to care: This was defined as a participant having reported, during follow-up contact, attending a clinic for HIV care subsequent to release. Participants who were unable to be contacted were considered not to have linked to care.
- Verified linkage to care: This was defined as a confirmation of self-reported linkage to care. Linkage was confirmed using abstracted clinic-based paper or electronic records.
- Lapse in ART possession: This was based on the self-reported date of first linkage to care and the self-reported days of ART provided at corrections release. A lapse was defined to have occurred if the time to first clinic visit from corrections release was greater than the reported medication supply received at corrections release. If there was no self-reported linkage to care a participant was also classified as having a lapse in ART possession.

#### Independent variables

We assessed independent variables for association with the primary outcome of self-reported linkage to care and the secondary outcome of verified linkage to care. We specifically included the following variables in bivariate modeling: sex, age group (< 35, ≥35 years old), CD4 count, duration of incarceration, location of HIV diagnosis, and location of ART initiation. The age group was divided at age 35 years based on a prior study from South Africa reporting generally lower linkage to care from community HIV testing among individuals < 35 years old [6]. Sex and age were selected as independent variables based on the evidence that each is associated with linkage to care. Incarceration duration and location of HIV diagnosis and ART initiation were included to assess for associations with corrections specific factors that may be associated with linkage to care. We were particularly interested in assessing whether place of ART initiation (while incarcerated or prior to incarceration) affected post-release linkage to care.

### Statistical analysis

We excluded from the primary outcome analysis participants who were known to have been re-incarcerated (consistent with prior analyses [15]), known to have died, or withdrew consent. Descriptive statistics were used to characterize the population.

Log-binomial regression was used to assess for bivariable and multivariable associations between self-reported linkage to care and verified linkage to care and independent variables. Log-binomial regression was selected to estimate relative risks for a common outcome. All regression analyses included adjustment for facility effect. We assessed the following independent variables: sex, age group (< 35, ≥35 years old), CD4 count closest to time of release, duration of incarceration, location of HIV diagnosis, and location of ART initiation. Bivariable analyses were initially performed. Independent variables with a *p* ≤ 0.1 in bivariable analyses were included in multivariable model building. Age group and correctional facility were included in all multivariable models regardless of *p* value. Sex was not included, a priori, in adjusted models due to the relatively small proportion of women in the study. Variables with *p* > 0.1 in multivariable modeling were eliminated in a stepwise manner starting with the variable with the highest *p* value. This process was continued until all selected variables had a *p* < 0.1. Through this process we developed final models for self-reported linkage to care and for verified linkage to care. Stata 14 was used for all analyses (STATA Corp. College Park, Texas, USA).

## Results

From May 2014 to December 2016, 608 inmates were screened for study eligibility and 554 (91%) met eligibility criteria. Reasons for ineligibility were not having any contact details for follow-up [6], not receiving HIV care [3], and unknown release date (45). Of the 554 who were eligible, 516 gave consent (93%). From these, 391 were released on ART. The majority of those not on ART at release were not eligible based on contemporaneous CD4 count criteria. Forty were subsequently excluded from analysis: 5 withdrew participation following release, 28 were known to be re-incarcerated, and 7 were known to have died. The remaining 351 contributed to the analyses. Overall, 301 (86%) participants were men, the median age was 35 years (interquartile range, [IQR]: 30, 40), and 266 (76%) were initiated on ART during the index incarceration (Table 1).

**Table 1:** Characteristics of the population

	n (%)
Sex	
Male	301 (86)
Female	50 (14)
Age group, years	
18–35	196 (56)
> 35	155 (44)
Country of origin	
South Africa	325 (92)
Lesotho	3 (1)
Mozambique	7 (2)
Zimbabwe	16 (4)
Place where received HIV diagnosis	
Correctional center	211 (60)
Not within justice system	134 (38)
Unknown	6 (2)
Started ART during index incarceration	
No	85 (24)
Yes	266 (76)
Duration of incarceration	
< 1 year	159 (44)
1–2 years	55 (16)
> 2 years	137 (39)
Last CD4 count prior to release, cells/mm^3^	
≤ 200	43 (20)
201–350	73 (33)
351–500	48 (22)
> 500	48 (22)
No CD4 count available	55 (25)
ART supply provided on release	
< 30 days	80 (23)
30–59 days	96 (27)
60–89 days	51 (14)
≥ 90 days	22 (6)
Missing	102 (29)

Follow-up contact was successful with the participant or household member for 257 (73%) of the participants. Linkage to care within 90 days was reported by 227 (65%). Based on reported ART supply at correctional facility release and timing of linkage to care, 164 (47%) participants had no lapse in ART supply. Verification of self-reported linkage to care was successful for 121 (34%). Among those with verified linkage to care within 90 days (for which we have the most precise linkage dates), 25% linked within 11 days, 50% within 28 days, and 75% within 49 days.

In bivariate log-binomial regression, adjusting for correctional facility, ART initiation during the current incarceration was associated with self-reported linkage to care (*p* = 0.03). Longer duration of incarceration had a trend toward association with self-reported linkage to care (*p* = 0.06; Table 2). In adjusted multivariable analysis including age group, longer duration of incarceration and ART initiation during the index incarceration each maintained significance or trended toward a significant association with increased linkage to care (Table 2).

**Table 2:** Participant characteristics and association with self-reported care transition (*n* = 351)

	n/total	bivariable risk ratio (95% confidence interval)^a^	multivariable risk ratio (95% confidence interval)^a^
Sex			
Male	191/301	Ref, *p* = 0.6	
Female	36/50	1.0 (0.86, .13)	
Age group, years			
18–35	121/196	Ref, *p* = 0.2	Ref, *p* = 0.4
> 35	106/155	1.1 (0.96, 1.3)	1.1 (0.91, 1.2)
Place where received HIV diagnosis			
Correctional center	130/211	Ref, *p* = 0.3	
Outside correctional center	93/134	1.1 (0.94, 1.3)	
Started ART during index incarceration			
No	46/83	Ref, *p* = 0.03	Ref, 0.06
Yes	181/268	1.2 (1.0, 1.5)	1.2 (0.99, 1.5)
Duration of incarceration			
< 1 year	97/159	Ref, p_trend_ = 0.03	Ref, *p* = 0.03
1–2 years	28/55	0.86 (0.65, 1.2)	0.60 (0.46, 0.77)
> 2 years	102/137	1.2 (1.0, 1.4)	1.1 (0.81, 1.3)
Last CD4 count prior to release, cells/mm^3^			
< =200	29/43	Ref, p_trend_ = 0.8	
201–350	51/73	1.0 (0.81, 1.3)	
351–500	30/48	0.95 (0.71, 1.3)	
> 500	38/55	1.1 (0.83, 1.4)	

^a^ Adjusted for correctional facility

The independent variable significantly associated with increased verified linkage to care was age > 35 years. Three additional variables trended toward association: HIV diagnosis outside of a correctional center, ART initiation during the index incarceration, and longer duration of incarceration (Table 3). In the final multivariable modeling, adjusted for correctional facility, older age maintained a significant association, site of HIV diagnosis and site of ART initiation maintained a trend toward significance, and duration of incarceration lost significance and was dropped from the model (Table 3).

**Table 3:** Participant characteristics and association with verified care transition (*n* = 351)

	n/total	bivariable risk ratio (95% confidence interval)^a^	multivariable risk ratio (95% confidence interval)^a^
Sex			
Male	105/301	Ref, *p* = 0.7	
Female	16/50	0.92 (0.57, 1.5)	
Age group, years			
18–35	55/196	Ref, *p* = 0.004	Ref, *p* = 0.004
> 35	66/155	1.5 (1.1, 2.0)	1.5 (1.1, 2.0)
Place where received HIV diagnosis			
Correctional center	65/211	Ref, *p* = 0.08	Ref, *p* = 0.1
Outside correctional center	53/134	1.3 (0.97, 1.7)	1.2 (0.93, 1.6)
Started ART during current incarceration			
No	22/83	Ref, *p* = 0.09	Ref, *p* = 0.1
Yes	99/268	1.4 (0.95, 2.1)	1.4 (0.92, 2.0)
Duration of incarceration			
< 1 year	48/159	Ref, p_trend_ = 0.1	
1–2 years	16/55	0.97 (0.60, 1.6)	
> 2 years	57/137	1.4 (0.99, 1.9)	
Last CD4 count prior to release, cells/mm^3^			
< =200	16/43	Ref, p_trend_ = 0.8	
201–350	24/73	0.61 (0.53, 1.4)	
351–500	21/48	1.2 (0.71, 2.0)	
> 500	19/55	0.94 (0.55, 1.6)	

^a^Adjusted for correctional facility

## Discussion

This is the first description from sub-Saharan Africa, of which we are aware, regarding the transition from HIV care in a correctional facility to community clinics. Linkage to care was self-reported by 65% of participants and could be verified with clinical documentation for 34%. At most, 47% of participants had no post-release lapse in ART supply. We observed greater self-reported linkage to care and a trend toward greater verified linkage to care among participants initiated on ART while in the correctional facility. These findings demonstrate an overall troubling gap in linkage to care, while also providing important re-assurance that initiating ART within correctional facilities does not exacerbate post-release loss from care.

One prior study from South Africa reported the proportion of community reentrants who continued care at the same off-site community clinic where they had received care during incarceration [9]. In that study, among 34 released inmates, 23 (68%) had at least one visit to the clinic post-release. The substantial changes that have occurred with HIV care in South Africa since the time of that study include expansion of ART services to correctional facilities and changes in ART initiation thresholds from a CD4 count < 200 cells/mm^3^ to universal treatment access [18–20]. Some of these changes may have effected care continuity.

Important research has been completed in high income countries on the transition from corrections to community care. Compared to some of these prior studies, our observed self-reported linkage to care was higher. Specifically, the proportion of ex-inmates linking to care in studies from the United States ranges from 28 to 95%; a median of 36% was calculated in a meta-analysis [10–15, 21–26]. There are several possible reasons for the potentially higher linkage to care that we observed. First, we may have over-estimated linkage to care through the use of self-report. Second, health insurance is generally necessary for accessing HIV care in the United States whereas HIV care is free within the public sector in South Africa. Third, substance use can interfere with linkage to care. While substance use is present in South Africa [27], it is likely more prevalent among the corrections population in the United States. Forth, the median duration of incarceration was longer in our study than many reports from the United States, a factor that has been previously associated with an improved care transition [28].

We noted increased verified linkage to care with older age and trends toward association with ART initiated during the index incarceration, HIV diagnosed outside of a correctional center, and longer duration of incarceration. Studies from the United States have consistently reported a similar association between older age and improved linkage to care [29–32]. Several studies have also reported a significant association between longer duration of incarceration and increased linkage to care [28, 29, 32]. One study from the United States reported a trend toward increased linkage to care with HIV diagnosis during the index incarceration in contrast to our finding [31]. We did not find any other studies that specifically addressed the timing of ART initiation and linkage to care during reentry.

This study has the strengths of recruiting participants while incarcerated and achieving enrollment of a high proportion of those who were eligible. Limitations include failure to determine outcomes for all participants due to the challenge with post-release follow-up. Participants whom we could not trace were more likely to lack a fixed address and not have regular contact with family members. We expect that this population was less likely to have engaged in care following release, although we cannot confirm this. Among those participants who we were able to contact, most reported clinic attendance. Of those reporting going to the clinic, we were only able to verify actual attendance for about 50%. There are several potential reasons for the low level of verification including over-reporting by participants due to the social desirability bias, a phenomenon reported in prior linkage to care studies [6, 33, 34]. It is also possible that we were unable to identify records for some participants who truly did link to care due to missing records (from electronic and paper clinic records), lack of laboratory testing (for querying electronic laboratory data), and use of pseudonyms by participants either in the study or at a clinic.

Additional considerations are limitations to generalizability. The small sample size and inclusion of correctional facilities in a single region in a single country limit the generalizability to more rural areas and beyond South Africa. In addition, we excluded individuals with expected release beyond the region of the study. It is plausible that linkage to care challenges were greater and linkage to care lower for those released to more distant locations.

## Conclusions

Innovative approaches are needed to monitor and support patients during the transition from correctional facilities to community HIV care in South Africa. Approaches may need to address individual level barriers through individual-level services and improve system level communication and patient monitoring between correctional health services and community clinics. Given the apparent attrition in HIV care during the corrections to community transition, further operational research is needed to better characterize this transition and identify effective approaches of retaining former inmates in care.

## Data Availability

The data set used for this publication is available upon request from the authors.
